# Gender job segregation

**DOI:** 10.47626/1679-4435-2023-1108

**Published:** 2023-11-24

**Authors:** Miguel Valencia-Contrera, Flérida Rivera-Rojas

**Affiliations:** 1 Universidad Andrés Bello, Facultad de Enfermería, Santiago, Región Metropolitana, Chile; 2 Universidad Católica del Maule, Departamento de Enfermería, Curicó, Región del Maule, Chile

Work has historically been characterized by having gender gaps that disfavor women’s participation in the development of different labor activities; among the wide range of associated phenomena, the present manuscript will address two of them: horizontal segregation and vertical segregation.

The phenomenon of horizontal segregation occurs when a labor market tends to show the predominance of the female image in certain fields and types of occupation, whereas vertical segregation occurs when there is scarce participation of women in management positions or in hierarchically higher positions within an organizational structure.

The European Institute for Gender Equality (EIGE) developed an index to measure the progress of this matter in the European Union and provide information on areas requiring improvement. In its 2021 report, the EIGE reported a small increase of only 0.2 points in the index compared to 2018, which indicates a regression in the annual progress.^1^ Furthermore, a low and almost stable score of 61.3 points was observed for the subdomain of segregation and quality of work in 2019, which is translated into a strong gender segregation in the labor market. In addition to this, the result of the COVID-19 pandemic, advances in terms of gender equality between groups could be halted or reversed.

In 2019 the International Labor Organization and the United Nations Development Programme published a report^2^ describing the reality of eight Latin American countries (Brazil, Colombia, Costa Rica, El Salvador, Uruguay, Mexico, Guatemala, and Ecuador), based on the Duncan Dissimilarity Index and on the Karmel and MacLachlan Index. This report observed that only El Salvador and Colombia experienced a decrease in the analyzed period (2000-2015); however, these were also the two countries that had the highest values at the beginning of the analyzed period.

In light of the foregoing, it is possible to state that there was, there is, and at least in the very near future, there will still be gender job segregation. In this scenario, the present authors emphasize the importance of focusing efforts on conducting studies to assess such segregation, because there is a scarcity of studies addressing this issue in the Latin American reality. Moreover, the scientific community is urged to study the health effects of gender segregation on the working population according to gender, considering the historically existing inequalities. Additionally, it is necessary to evaluate each gender-associated role in the reality of each country, which lead to the creation of more equitable public policies and health programs adapted to the reality of each person, whose need has been manifested by the scientific community.^3^
